# Gene capture prediction and overlap estimation in EST sequencing from one or multiple libraries

**DOI:** 10.1186/1471-2105-6-300

**Published:** 2005-12-13

**Authors:** Ji-Ping Z Wang, Bruce G Lindsay, Liying Cui, P Kerr Wall, Josh Marion, Jiaxuan Zhang, Claude W dePamphilis

**Affiliations:** 1 Department of Statistics, Northwestern University, Evanston, IL 60208, USA; 2 Department of Statistics, Penn State University, University Park 16802, USA; 3 Department of Biology, Penn State University, University Park 16802, USA; 4 Department of Computer Science, Penn State University, University Park 16802, USA; 5 College of Software, Tsinghua University, Beijing, 100086, PR China

## Abstract

**Background:**

In expressed sequence tag (EST) sequencing, we are often interested in how many genes we can capture in an EST sample of a targeted size. This information provides insights to sequencing efficiency in experimental design, as well as clues to the diversity of expressed genes in the tissue from which the library was constructed.

**Results:**

We propose a compound Poisson process model that can accurately predict the gene capture in a future EST sample based on an initial EST sample. It also allows estimation of the number of expressed genes in one cDNA library or co-expressed in two cDNA libraries. The superior performance of the new prediction method over an existing approach is established by a simulation study. Our analysis of four *Arabidopsis thaliana *EST sets suggests that the number of expressed genes present in four different cDNA libraries of *Arabidopsis thaliana *varies from 9155 (root) to 12005 (silique). An observed fraction of co-expressed genes in two different EST sets as low as 25% can correspond to an actual overlap fraction greater than 65%.

**Conclusion:**

The proposed method provides a convenient tool for gene capture prediction and cDNA library property diagnosis in EST sequencing.

## Background

An expressed sequence tag (EST) set surveys a cDNA library for two important types of information: the transcript sequence and transcript abundance [1]. Both of these can be obtained through EST clustering, a process that identifies and assembles sibling ESTs (ESTs from the same gene) [2-8]. The assembly of ESTs in each cluster is a partially or completely restored transcript (if there is no clustering error), and the number of ESTs within each cluster then represents the abundance of this transcript or mRNA species in the cDNA library. The sequence information has greatly facilitated numerous applications in genomic research including the construction of gene indexing systems, novel gene discovery, genome annotation, SNP typing, splicing detection and microarray probe design [9-18]. The transcript abundance information conveyed by the EST data has been used for gene expression differentiation and gene discovery rate estimation [19-21].

In this paper we consider multiple applications that require modeling of the expression data for inference of cDNA library properties. Key questions of interest include, (a) how many new genes can be captured in an additional sample of a targeted size based on the current EST data from the same library? (b) how many genes are expressed in one tissue or multiple tissues given the EST data? and (c) how many genes are co-expressed in two tissues? Answers to these questions, we believe, will provide not only new clues to the diversity of expressed genes in a wide diversity of organisms that have been subject to EST sequencing, but also a way to predict sequencing outcomes. For example, the overlap of expressed genes can be indicative of functional similarity of two tissues; the expected gene capture from an additional sample can be useful for budgeting future sequencing efforts.

As "expression evidence", EST data already plays a crucial role in gene annotation and inference of the number of expressed genes in the transcriptome of an organism [22-25]. However two major challenges exist in direct estimation of gene capture or the total number of genes expressed in a tissue based on EST data alone. The first challenge arises from EST clustering error. Errors from different sources can bias the number of observed genes upward by 35% – 40% [25-27]. For 5' ESTs, the false separation error is especially problematic; insufficient overlap between sibling ESTs (ESTs from the same gene) can explain a fraction up to 80% of these clustering errors [27]. In this paper, the gene cluster profile data (defined below) for 5' ESTs was obtained after correcting for insufficient overlap error (ISO error) using the method introduced in [27].

Given that good data has been generated from EST clustering, it remains a challenge to make accurate predictions of gene capture that will be expected in future sequencing experiments. Question (a) was recently addressed by [21] where prediction of gene capture in an additional sample of size larger than the initial sample requires parametric fitting of the transcript abundance distribution to avoid wild variability of the estimator (i.e., data are fit to a Negative Binomial model derived from a Poisson-Gamma setting that allows the α parameter in the Gamma to be < 0, see also [28,29]). However an inappropriate assumption of the transcript abundance distribution (Gamma here) could result in systematic bias in estimation [30]. The performance of this approach in the EST problem has yet been well established.

In this paper we propose a compound Poisson process approach for accurate prediction of gene capture in EST sequencing. The superior performance of the new prediction method over the existing method implemented by [21] in a computer program *egene *is established with a simulation study. We discuss how this method can be applied to estimate the number of genes expressed in one cDNA library, or co-expressed in two libraries. Finally we illustrate the new prediction method with four EST sets from the flowering plant *Arabidopsis thaliana*.

## Results and Discussion

### Compound Poisson process model

Let *N *be the number of genes represented with transcripts in the cDNA library. **X **= {*X*_1_, ..., *X_N_*} will be the number of tags observed from each distinct gene species. If gene *i *is not captured in the EST sample, then *X_i _*= 0. Let nj=∑i=1NI(Xi=j)
 MathType@MTEF@5@5@+=feaafiart1ev1aaatCvAUfKttLearuWrP9MDH5MBPbIqV92AaeXatLxBI9gBaebbnrfifHhDYfgasaacH8akY=wiFfYdH8Gipec8Eeeu0xXdbba9frFj0=OqFfea0dXdd9vqai=hGuQ8kuc9pgc9s8qqaq=dirpe0xb9q8qiLsFr0=vr0=vr0dc8meaabaqaciGacaGaaeqabaqabeGadaaakeaacqWGUbGBdaWgaaWcbaGaemOAaOgabeaakiabg2da9maaqadabaGaemysaK0aaeWaaeaacqWGybawdaWgaaWcbaGaemyAaKgabeaakiabg2da9iabdQgaQbGaayjkaiaawMcaaaWcbaGaemyAaKMaeyypa0JaeGymaedabaGaemOta4eaniabggHiLdaaaa@3EF5@, for *j *= 0,1, ......, be the number of genes that had *j *ESTs in the sample, *D *= ∑*_j>0 _n_j _*be the observed total and *S *= ∑*_j>0 _jn_j _*be the current EST sample size. Estimation of *N *is equivalent to estimation of the zero class size *n*_0_. We call the summary data **n **= {*n*_1_, *n*_2_, ...} *gene cluster profile *data.

Let *p*_*i *_be the transcript abundance for gene *i*, i.e. ∑i=1Npi=1
 MathType@MTEF@5@5@+=feaafiart1ev1aaatCvAUfKttLearuWrP9MDH5MBPbIqV92AaeXatLxBI9gBaebbnrfifHhDYfgasaacH8akY=wiFfYdH8Gipec8Eeeu0xXdbba9frFj0=OqFfea0dXdd9vqai=hGuQ8kuc9pgc9s8qqaq=dirpe0xb9q8qiLsFr0=vr0=vr0dc8meaabaqaciaacaGaaeqabaqabeGadaaakeaadaaeWaqaaiabdchaWnaaBaaaleaacqWGPbqAaeqaaOGaeyypa0JaeGymaedaleaacqWGPbqAcqGH9aqpcqaIXaqmaeaacqWGobGta0GaeyyeIuoaaaa@3814@. The capture of ESTs from each gene in EST sequencing can be regarded as a Poisson process where the EST sample size *S *measures the "time" and *p*_*i *_plays the role of Poisson mean parameter rate, i.e., the probability of observing *x*_*i *_ESTs from gene *i *equals f(xi;S,pi)=e−Spi(Spi)xixi!
 MathType@MTEF@5@5@+=feaafiart1ev1aaatCvAUfKttLearuWrP9MDH5MBPbIqV92AaeXatLxBI9gBaebbnrfifHhDYfgasaacH8akY=wiFfYdH8Gipec8Eeeu0xXdbba9frFj0=OqFfea0dXdd9vqai=hGuQ8kuc9pgc9s8qqaq=dirpe0xb9q8qiLsFr0=vr0=vr0dc8meaabaqaciGacaGaaeqabaqabeGadaaakeaacqWGMbGzdaqadaqaaiabdIha4naaBaaaleaacqWGPbqAaeqaaOGaei4oaSJaem4uamLaeiilaWIaemiCaa3aaSbaaSqaaiabdMgaPbqabaaakiaawIcacaGLPaaacqGH9aqpdaWcaaqaaiabdwgaLnaaCaaaleqabaGaeyOeI0Iaem4uamLaemiCaa3aaSbaaWqaaiabdMgaPbqabaaaaOWaaeWaaeaacqWGtbWucqWGWbaCdaWgaaWcbaGaemyAaKgabeaaaOGaayjkaiaawMcaamaaCaaaleqabaGaemiEaG3aaSbaaWqaaiabdMgaPbqabaaaaaGcbaGaemiEaG3aaSbaaSqaaiabdMgaPbqabaGccqGGHaqiaaaaaa@4D08@. The Poisson distribution can be regarded as an approximation to the actual Binomial distribution *Bin*(*S, p*_*i*_) for a large *S *and a tiny *p*_*i *_[31]. Without loss of generality, we would treat the current sample size as one unit time, and let λ_*i *_= *Sp*_*i*_. Hence sampling an additional *S*_1 _ESTs corresponds to a Poisson process on time interval [1, 1+t] where *t *= *S*_1_*/S*. Considering substantial heterogeneity in the transcript abundance *p*_*i *_(and hence λ_*i*_), we further assume that λ_*i *_follows an unknown non-degenerate distribution *Q*(λ). The marginal distribution of *X *then follows a compound Poisson process [29,32], i.e.

f(x;Q)=∫e−λλxx! dQ(λ).
 MathType@MTEF@5@5@+=feaafiart1ev1aaatCvAUfKttLearuWrP9MDH5MBPbIqV92AaeXatLxBI9gBaebbnrfifHhDYfgasaacH8akY=wiFfYdH8Gipec8Eeeu0xXdbba9frFj0=OqFfea0dXdd9vqai=hGuQ8kuc9pgc9s8qqaq=dirpe0xb9q8qiLsFr0=vr0=vr0dc8meaabaqaciGacaGaaeqabaqabeGadaaakeaacqWGMbGzdaqadaqaaiabdIha4jabcUda7iabdgfarbGaayjkaiaawMcaaiabg2da9maapeaabaWaaSaaaeaacqWGLbqzdaahaaWcbeqaaiabgkHiTGGaaiab=T7aSbaakiab=T7aSnaaCaaaleqabaGaemiEaGhaaaGcbaGaemiEaGNaeiyiaecaaaWcbeqab0Gaey4kIipakiaaykW7cqWGKbazcqWGrbqudaqadaqaaiab=T7aSbGaayjkaiaawMcaaiabc6caUaaa@483E@

Let *D *be the number of distinct genes captured on the Poisson process [0, 1] and *D*_*t *_be the additional distinct genes captured on [1, 1+t], then (*D, D*_*t*_) has a Multinomial distribution as follows

f(D,Dt;N,Q)=(ND,Dt)q1DqtDt(1−q1−qt)N−D−Dt,     (1)
 MathType@MTEF@5@5@+=feaafiart1ev1aaatCvAUfKttLearuWrP9MDH5MBPbIqV92AaeXatLxBI9gBaebbnrfifHhDYfgasaacH8akY=wiFfYdH8Gipec8Eeeu0dXdbba9frFj0=OqFfea0dXdd9vqai=hGuQ8kuc9pgc9s8qqaq=dirpe0xb9q8qiLsFr0=vr0=vr0dc8meaabaqaciGacaGaaeqabaqabeGadaaakeaacqWGMbGzdaqadaqaaiabdseaejabcYcaSiabdseaenaaBaaaleaacqWG0baDaeqaaOGaei4oaSJaemOta4KaeiilaWIaemyuaefacaGLOaGaayzkaaGaeyypa0ZaaeWaaeaafaqabeGabaaabaGaemOta4eabaGaemiraqKaeiilaWIaemiraq0aaSbaaSqaaiabdsha0bqabaaaaaGccaGLOaGaayzkaaGaemyCae3aa0baaSqaaiabigdaXaqaaiabdseaebaakiabdghaXnaaDaaaleaacqWG0baDaeaacqWGebardaWgaaadbaGaemiDaqhabeaaaaGcdaqadaqaaiabigdaXiabgkHiTiabdghaXnaaBaaaleaacqaIXaqmaeqaaOGaeyOeI0IaemyCae3aaSbaaSqaaiabdsha0bqabaaakiaawIcacaGLPaaadaahaaWcbeqaaiabd6eaojabgkHiTiabdseaejabgkHiTiabdseaenaaBaaameaacqWG0baDaeqaaaaakiabcYcaSiaaxMaacaWLjaGaeiikaGIaeGymaeJaeiykaKcaaa@5FDF@

where

*q*_1 _≡ *q*_1_(*Q*) = ∫ (1 - *e*^-λ^)*dQ*(λ), *q_t _*≡ *q_t_*(*Q*) = ∫ *e*^-λ^(1 - *e*^-*t*λ^)*dQ*(λ).

In words, *q*_1 _is the probability of observing at least one tag from a random gene on [0, 1], and *q*_*t *_is that of observing zero tags on [0, 1] but at least 1 tag on [1, 1+t].

In the EST problem, one focal interest is the expectation of additional distinct genes that can be captured in the time period [1, 1 *+t*] given the current EST data. The distribution form in equation (1) implies that the conditional capture *D*_*t *_given the current sample only depends on *D*. More explicitly, the conditional distribution of *D*_*t*_|*D *is a Binomial (*N *- *D*, qt1−q1
 MathType@MTEF@5@5@+=feaafiart1ev1aaatCvAUfKttLearuWrP9MDH5MBPbIqV92AaeXatLxBI9gBaebbnrfifHhDYfgasaacH8akY=wiFfYdH8Gipec8Eeeu0xXdbba9frFj0=OqFfea0dXdd9vqai=hGuQ8kuc9pgc9s8qqaq=dirpe0xb9q8qiLsFr0=vr0=vr0dc8meaabaqaciaacaGaaeqabaqabeGadaaakeaadaWcaaqaaiabdghaXnaaBaaaleaacqWG0baDaeqaaaGcbaGaeGymaeJaeyOeI0IaemyCae3aaSbaaSqaaiabigdaXaqabaaaaaaa@3432@), and hence

E(Dt|D)=(N−D)qt1−q1.     (2)
 MathType@MTEF@5@5@+=feaafiart1ev1aaatCvAUfKttLearuWrP9MDH5MBPbIqV92AaeXatLxBI9gBaebbnrfifHhDYfgasaacH8akY=wiFfYdH8Gipec8Eeeu0xXdbba9frFj0=OqFfea0dXdd9vqai=hGuQ8kuc9pgc9s8qqaq=dirpe0xb9q8qiLsFr0=vr0=vr0dc8meaabaqaciGacaGaaeqabaqabeGadaaakeaacqWGfbqrdaqadaqaaiabdseaenaaBaaaleaacqWG0baDaeqaaOGaeiiFaWNaemiraqeacaGLOaGaayzkaaGaeyypa0ZaaeWaaeaacqWGobGtcqGHsislcqWGebaraiaawIcacaGLPaaadaWcaaqaaiabdghaXnaaBaaaleaacqWG0baDaeqaaaGcbaGaeGymaeJaeyOeI0IaemyCae3aaSbaaSqaaiabigdaXaqabaaaaOGaeiOla4IaaCzcaiaaxMaacqGGOaakcqaIYaGmcqGGPaqkaaa@46A1@

To calculate the expectation, one needs to estimate *N *and *Q *first. If *Q *is known, we have

*E*(*D*) = *Nq*_1_.

The observed total *D *is a natural estimate of *E*(*D*). The maximum likelihood estimator of *N *is N^=Dq1
 MathType@MTEF@5@5@+=feaafiart1ev1aaatCvAUfKttLearuWrP9MDH5MBPbIqV92AaeXatLxBI9gBaebbnrfifHhDYfgasaacH8akY=wiFfYdH8Gipec8Eeeu0xXdbba9frFj0=OqFfea0dXdd9vqai=hGuQ8kuc9pgc9s8qqaq=dirpe0xb9q8qiLsFr0=vr0=vr0dc8meaabaqaciGacaGaaeqabaqabeGadaaakeaacuWGobGtgaqcaiabg2da9maalaaabaGaemiraqeabaGaemyCae3aaSbaaSqaaiabigdaXaqabaaaaaaa@3291@[33]. Since *Q *is unknown, we can obtain an estimate Q^
 MathType@MTEF@5@5@+=feaafiart1ev1aaatCvAUfKttLearuWrP9MDH5MBPbIqV92AaeXatLxBI9gBaebbnrfifHhDYfgasaacH8akY=wiFfYdH8Gipec8Eeeu0xXdbba9frFj0=OqFfea0dXdd9vqai=hGuQ8kuc9pgc9s8qqaq=dirpe0xb9q8qiLsFr0=vr0=vr0dc8meaabaqaciGacaGaaeqabaqabeGadaaakeaacuWGrbqugaqcaaaa@2DE9@ by nonparametric maximum likelihood estimation (see Methods). Replacing *q*_1_, *q*_*t *_by q^1≡q1(Q^),q^t≡qt(Q^)
 MathType@MTEF@5@5@+=feaafiart1ev1aaatCvAUfKttLearuWrP9MDH5MBPbIqV92AaeXatLxBI9gBaebbnrfifHhDYfgasaacH8akY=wiFfYdH8Gipec8Eeeu0xXdbba9frFj0=OqFfea0dXdd9vqai=hGuQ8kuc9pgc9s8qqaq=dirpe0xb9q8qiLsFr0=vr0=vr0dc8meaabaqaciGacaGaaeqabaqabeGadaaakeaacuWGXbqCgaqcamaaBaaaleaacqaIXaqmaeqaaOGaeyyyIORaemyCae3aaSbaaSqaaiabigdaXaqabaGcdaqadaqaaiqbdgfarzaajaaacaGLOaGaayzkaaGaeiilaWIafmyCaeNbaKaadaWgaaWcbaGaemiDaqhabeaakiabggMi6kabdghaXnaaBaaaleaacqWG0baDaeqaaOWaaeWaaeaacuWGrbqugaqcaaGaayjkaiaawMcaaaaa@420E@ and *N *by N^=Dq^1
 MathType@MTEF@5@5@+=feaafiart1ev1aaatCvAUfKttLearuWrP9MDH5MBPbIqV92AaeXatLxBI9gBaebbnrfifHhDYfgasaacH8akY=wiFfYdH8Gipec8Eeeu0xXdbba9frFj0=OqFfea0dXdd9vqai=hGuQ8kuc9pgc9s8qqaq=dirpe0xb9q8qiLsFr0=vr0=vr0dc8meaabaqaciGacaGaaeqabaqabeGadaaakeaacuWGobGtgaqcaiabg2da9maalaaabaGaemiraqeabaGafmyCaeNbaKaadaWgaaWcbaGaeGymaedabeaaaaaaaa@32A1@ in (2) gives an estimator of *E*(*D*_*t*_|*D*) as

E(Dt|D)_=(Dq^1−D)q^t1−q^1=Dq^tq^1
 MathType@MTEF@5@5@+=feaafiart1ev1aaatCvAUfKttLearuWrP9MDH5MBPbIqV92AaeXatLxBI9gBaebbnrfifHhDYfgasaacH8akY=wiFfYdH8Gipec8Eeeu0xXdbba9frFj0=OqFfea0dXdd9vqai=hGuQ8kuc9pgc9s8qqaq=dirpe0xb9q8qiLsFr0=vr0=vr0dc8meaabaqaciaacaGaaeqabaqabeGadaaakeaadaqiaaqaaiabdweafnaabmaabaGaemiraq0aaSbaaSqaaiabdsha0bqabaGccqGG8baFcqWGebaraiaawIcacaGLPaaaaiaawkWaaiabg2da9iabcIcaOmaalaaabaGaemiraqeabaGafmyCaeNbaKaadaWgaaWcbaGaeGymaedabeaaaaGccqGHsislcqWGebarcqGGPaqkdaWcaaqaaiqbdghaXzaajaWaaSbaaSqaaiabdsha0bqabaaakeaacqaIXaqmcqGHsislcuWGXbqCgaqcamaaBaaaleaacqaIXaqmaeqaaaaakiabg2da9iabdseaenaalaaabaGafmyCaeNbaKaadaWgaaWcbaGaemiDaqhabeaaaOqaaiqbdghaXzaajaWaaSbaaSqaaiabigdaXaqabaaaaaaa@4D5B@.

From a different perspective, since *E*(*D*_*t*_) = *Nq*_*t*_, replacing *N *by N^=Dq^1
 MathType@MTEF@5@5@+=feaafiart1ev1aaatCvAUfKttLearuWrP9MDH5MBPbIqV92AaeXatLxBI9gBaebbnrfifHhDYfgasaacH8akY=wiFfYdH8Gipec8Eeeu0xXdbba9frFj0=OqFfea0dXdd9vqai=hGuQ8kuc9pgc9s8qqaq=dirpe0xb9q8qiLsFr0=vr0=vr0dc8meaabaqaciGacaGaaeqabaqabeGadaaakeaacuWGobGtgaqcaiabg2da9maalaaabaGaemiraqeabaGafmyCaeNbaKaadaWgaaWcbaGaeGymaedabeaaaaaaaa@32A1@ and *q*_*t *_by q^t
 MathType@MTEF@5@5@+=feaafiart1ev1aaatCvAUfKttLearuWrP9MDH5MBPbIqV92AaeXatLxBI9gBaebbnrfifHhDYfgasaacH8akY=wiFfYdH8Gipec8Eeeu0xXdbba9frFj0=OqFfea0dXdd9vqai=hGuQ8kuc9pgc9s8qqaq=dirpe0xb9q8qiLsFr0=vr0=vr0dc8meaabaqaciGacaGaaeqabaqabeGadaaakeaacuWGXbqCgaqcamaaBaaaleaacqWG0baDaeqaaaaa@2FC6@ gives an estimator of the unconditional mean *E*(*D_t_*) as

E(Dt)_=Dq^tq^1,
 MathType@MTEF@5@5@+=feaafiart1ev1aaatCvAUfKttLearuWrP9MDH5MBPbIqV92AaeXatLxBI9gBaebbnrfifHhDYfgasaacH8akY=wiFfYdH8Gipec8Eeeu0xXdbba9frFj0=OqFfea0dXdd9vqai=hGuQ8kuc9pgc9s8qqaq=dirpe0xb9q8qiLsFr0=vr0=vr0dc8meaabaqaciGacaGaaeqabaqabeGadaaakeaadaqiaaqaaiabdweafnaabmaabaGaemiraq0aaSbaaSqaaiabdsha0bqabaaakiaawIcacaGLPaaaaiaawkWaaiabg2da9iabdseaenaalaaabaGafmyCaeNbaKaadaWgaaWcbaGaemiDaqhabeaaaOqaaiqbdghaXzaajaWaaSbaaSqaaiabigdaXaqabaaaaOGaeiilaWcaaa@3B8E@

which is the same as E(Dt|D)_
 MathType@MTEF@5@5@+=feaafiart1ev1aaatCvAUfKttLearuWrP9MDH5MBPbIqV92AaeXatLxBI9gBaebbnrfifHhDYfgasaacH8akY=wiFfYdH8Gipec8Eeeu0xXdbba9frFj0=OqFfea0dXdd9vqai=hGuQ8kuc9pgc9s8qqaq=dirpe0xb9q8qiLsFr0=vr0=vr0dc8meaabaqaciGacaGaaeqabaqabeGadaaakeaadaqiaaqaaiabdweafnaabmaabaGaemiraq0aaSbaaSqaaiabdsha0bqabaGccqGG8baFcqWGebaraiaawIcacaGLPaaaaiaawkWaaaaa@3555@ derived above. In other words, the quantity Dq^tq^1
 MathType@MTEF@5@5@+=feaafiart1ev1aaatCvAUfKttLearuWrP9MDH5MBPbIqV92AaeXatLxBI9gBaebbnrfifHhDYfgasaacH8akY=wiFfYdH8Gipec8Eeeu0xXdbba9frFj0=OqFfea0dXdd9vqai=hGuQ8kuc9pgc9s8qqaq=dirpe0xb9q8qiLsFr0=vr0=vr0dc8meaabaqaciGacaGaaeqabaqabeGadaaakeaacqWGebardaWcaaqaaiqbdghaXzaajaWaaSbaaSqaaiabdsha0bqabaaakeaacuWGXbqCgaqcamaaBaaaleaacqaIXaqmaeqaaaaaaaa@3388@ can be used as an estimator for either the conditional or unconditional mean. In the simulation study section, we will investigate the performance of this estimator with respect to these two roles.

To measure the sequencing efficiency, we define the *expected sequencing redundancy *ρ as the average EST count per gene. An estimate of ρ at time 1 + *t *would be



The methods for *Q *estimation, confidence interval construction and cDNA library overlap estimation are presented in METHODS.

### Simulation studies

#### Estimating unconditional mean *E*(*D_t_*)

To investigate the performance of the proposed compound Poisson process method (to be called CPP below) as an unconditional mean estimator, we created three pseudo cDNA libraries from the following three settings: (I) *N *= 5000 and the transcript abundance followed a log normal distribution as f(pi)=e−[log(pi)−2]2/22π(pi)
 MathType@MTEF@5@5@+=feaafiart1ev1aaatCvAUfKttLearuWrP9MDH5MBPbIqV92AaeXatLxBI9gBaebbnrfifHhDYfgasaacH8akY=wiFfYdH8Gipec8Eeeu0xXdbba9frFj0=OqFfea0dXdd9vqai=hGuQ8kuc9pgc9s8qqaq=dirpe0xb9q8qiLsFr0=vr0=vr0dc8meaabaqaciaacaGaaeqabaqabeGadaaakeaacqWGMbGzdaqadaqaaiabdchaWnaaBaaaleaacqWGPbqAaeqaaaGccaGLOaGaayzkaaGaeyypa0ZaaSaaaeaacqWGLbqzdaahaaWcbeqaaiabgkHiTmaadmaabaWexLMBbXgBcf2CPn2qVrwzqf2zLnharyGvLjhzH5wyaGabciaa=XgacaWFVbGaa83zamaabmaabaGaemiCaa3aaSbaaWqaaiabdMgaPbqabaaaliaawIcacaGLPaaacqGHsislcqaIYaGmaiaawUfacaGLDbaadaahaaadbeqaaiabikdaYaaaliabc+caViabikdaYaaaaOqaamaakaaabaGaeGOmaiJaeqiWdahaleqaaOWaaeWaaeaacqWGWbaCdaWgaaWcbaGaemyAaKgabeaaaOGaayjkaiaawMcaaaaaaaa@5613@; (II) *N *= 10000 and *p*_*i *_had an exponential distribution with mean 0.5, i.e. *f*(*pi*) = 2e^-2*pi*^*; *and (III) *N *= 10000 and *p*_*i *_had a gamma distribution with α = 0.2, β = 3, i.e. f(pi)=30.2Γ(0.2)pi−0.8e−3pi
 MathType@MTEF@5@5@+=feaafiart1ev1aaatCvAUfKttLearuWrP9MDH5MBPbIqV92AaeXatLxBI9gBaebbnrfifHhDYfgasaacH8akY=wiFfYdH8Gipec8Eeeu0xXdbba9frFj0=OqFfea0dXdd9vqai=hGuQ8kuc9pgc9s8qqaq=dirpe0xb9q8qiLsFr0=vr0=vr0dc8meaabaqaciaacaGaaeqabaqabeGadaaakeaacqWGMbGzdaqadaqaaiabdchaWnaaBaaaleaacqWGPbqAaeqaaaGccaGLOaGaayzkaaGaeyypa0ZaaSaaaeaacqaIZaWmdaahaaWcbeqaaiabicdaWiabc6caUiabikdaYaaaaOqaaiabfo5ahnaabmaabaGaeGimaaJaeiOla4IaeGOmaidacaGLOaGaayzkaaaaaiabdchaWnaaDaaaleaacqWGPbqAaeaacqGHsislcqaIWaamcqGGUaGlcqaI4aaoaaGccqWGLbqzdaahaaWcbeqaaiabgkHiTiabiodaZiabdchaWnaaBaaameaacqWGPbqAaeqaaaaaaaa@4A48@.

**Figure 1:**
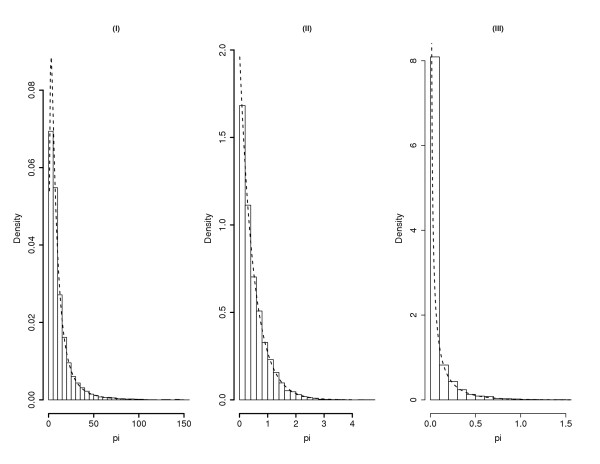
**Relative abundance distributions of mRNA transcripts in the simulation. **(I) log normal: f(pi)=e−[log(pi)−2]2/22π(pi)
 MathType@MTEF@5@5@+=feaafiart1ev1aaatCvAUfKttLearuWrP9MDH5MBPbIqV92AaeXatLxBI9gBaebbnrfifHhDYfgasaacH8akY=wiFfYdH8Gipec8Eeeu0xXdbba9frFj0=OqFfea0dXdd9vqai=hGuQ8kuc9pgc9s8qqaq=dirpe0xb9q8qiLsFr0=vr0=vr0dc8meaabaqaciaacaGaaeqabaqabeGadaaakeaacqWGMbGzdaqadaqaaiabdchaWnaaBaaaleaacqWGPbqAaeqaaaGccaGLOaGaayzkaaGaeyypa0ZaaSaaaeaacqWGLbqzdaahaaWcbeqaaiabgkHiTmaadmaabaWexLMBbXgBcf2CPn2qVrwzqf2zLnharyGvLjhzH5wyaGabciaa=XgacaWFVbGaa83zamaabmaabaGaemiCaa3aaSbaaWqaaiabdMgaPbqabaaaliaawIcacaGLPaaacqGHsislcqaIYaGmaiaawUfacaGLDbaadaahaaadbeqaaiabikdaYaaaliabc+caViabikdaYaaaaOqaamaakaaabaGaeGOmaiJaeqiWdahaleqaaOWaaeWaaeaacqWGWbaCdaWgaaWcbaGaemyAaKgabeaaaOGaayjkaiaawMcaaaaaaaa@5613@ (II) exponential: f(pi)=2e−2pi
 MathType@MTEF@5@5@+=feaafiart1ev1aaatCvAUfKttLearuWrP9MDH5MBPbIqV92AaeXatLxBI9gBaebbnrfifHhDYfgasaacH8akY=wiFfYdH8Gipec8Eeeu0xXdbba9frFj0=OqFfea0dXdd9vqai=hGuQ8kuc9pgc9s8qqaq=dirpe0xb9q8qiLsFr0=vr0=vr0dc8meaabaqaciaacaGaaeqabaqabeGadaaakeaacqWGMbGzdaqadaqaaiabdchaWnaaBaaaleaacqWGPbqAaeqaaaGccaGLOaGaayzkaaGaeyypa0JaeGOmaiJaemyzau2aaWbaaSqabeaacqGHsislcqaIYaGmcqWGWbaCdaWgaaadbaGaemyAaKgabeaaaaaaaa@3ACC@ and (III) gamma: f(pi)=30.2Γ(0.2)pi−0.8e−3pi
 MathType@MTEF@5@5@+=feaafiart1ev1aaatCvAUfKttLearuWrP9MDH5MBPbIqV92AaeXatLxBI9gBaebbnrfifHhDYfgasaacH8akY=wiFfYdH8Gipec8Eeeu0xXdbba9frFj0=OqFfea0dXdd9vqai=hGuQ8kuc9pgc9s8qqaq=dirpe0xb9q8qiLsFr0=vr0=vr0dc8meaabaqaciaacaGaaeqabaqabeGadaaakeaacqWGMbGzdaqadaqaaiabdchaWnaaBaaaleaacqWGPbqAaeqaaaGccaGLOaGaayzkaaGaeyypa0ZaaSaaaeaacqaIZaWmdaahaaWcbeqaaiabicdaWiabc6caUiabikdaYaaaaOqaaiabfo5ahnaabmaabaGaeGimaaJaeiOla4IaeGOmaidacaGLOaGaayzkaaaaaiabdchaWnaaDaaaleaacqWGPbqAaeaacqGHsislcqaIWaamcqGGUaGlcqaI4aaoaaGccqWGLbqzdaahaaWcbeqaaiabgkHiTiabiodaZiabdchaWnaaBaaameaacqWGPbqAaeqaaaaaaaa@4A48@

Two hundred Monte-Carlo samples were drawn from each setting with sample size S = 3000 for (I), S = 6000 for (II) and S = 5000 for (III) according to the relative abundance of the transcripts, i.e. pi∑i=1Npi
 MathType@MTEF@5@5@+=feaafiart1ev1aaatCvAUfKttLearuWrP9MDH5MBPbIqV92AaeXatLxBI9gBaebbnrfifHhDYfgasaacH8akY=wiFfYdH8Gipec8Eeeu0xXdbba9frFj0=OqFfea0dXdd9vqai=hGuQ8kuc9pgc9s8qqaq=dirpe0xb9q8qiLsFr0=vr0=vr0dc8meaabaqaciaacaGaaeqabaqabeGadaaakeaadaWcaaqaaiabdchaWnaaBaaaleaacqWGPbqAaeqaaaGcbaWaaabmaeaacqWGWbaCdaWgaaWcbaGaemyAaKgabeaaaeaacqWGPbqAcqGH9aqpcqaIXaqmaeaacqWGobGta0GaeyyeIuoaaaaaaa@3913@ These three distributions are all rightward skewed (See Figure 1), which appears to be a reasonable characterization of the expression pattern as observed from most EST data sets. The results from the CPP method are compared in Table 1 with the existing nonparametric empirical Bayes method due to [29,34], (which has been implemented by Susko and Roger [21] in the EST data analysis program *egene *available at [35] (to be called the SR method below).

**Table 1:** Comparing CPP method with nonparametric eB method in estimation of the unconditional mean *E*(*D*_*t*_). The theoretical unconditional mean at *t *was calculated based on the compound Poisson process model, i.e. *E*(*D*_*t*_) = *Nq*_*t *_where *q*_*t *_was calculated based on the CPP model. The entries in the row of CPP or SR are the Mean and root of Mean Squared Error(rMSE) (in parentheses) based on 200 Monte Carlo samples. A -(-) indicates that the mean or rMSE was not calculated because of extremely large or negative estimates from the SR method. For (I), *N q*_1 _and *S *were 5000, 0.36 and 3000; for (II), 10000, 0.375, 6000, and for (III) 10000, 0.221, 5000 respectively.

	t	0.5	1	1.5	2
(I)	*E*(*D*_*t*_)	497	873	1168	1406
	CPP	500(16.4)	873(35.6)	1160(58.8)	1386(85.8)
	SR	501(17.3)	877(43)	-(-)	-(-)

(II)	*E*(*D*_*t*_)	988	1707	2253	2682
	CPP	985(21.4)	1697(48.8)	2230(83.7)	2639(125.6)
	SR	985(22.1)	1698(58.4)	2218(183.3)	-(-)

(III)	*E(D*_*t*_*]*	464	801	1062	1273
	CPP	462(15.9)	793(36.5)	1045(62.5)	1242(93.5)
	SR	463(16.7)	799(45.2)	-(-)	-(-)

The simulations under the three different transcript abundance distributions reached very similar conclusions. The CPP method provides very reliable estimates for *t *≤ 2 while the SR method only works well for *t *≤ 1 (but less precise than the CPP method in terms of rMSE). When *t *≤ 1, the SR method cannot be recommended because it frequently produced negative or extremely variable estimates.

#### Estimating conditional mean *E*(*D*_*t*_|*D*)

Since our focal interest is the additional distinct genes that can be captured over the time period [1, 1 + *t*] conditioned on the current capture *D*, i.e. *E*(*D*_*t*_|*D*), we now investigate the performance of the CPP method for this end based on two typical EST samples simulated from situation (I) and (II).

The first EST set was simulated from situation (I) at sample size *S *= 3000. The resulting gene cluster profile data was **n **= (*n*_1_...*n*_10_) = (1162, 392, 170, 63, 21, 12, 8, 5, 1, 1), and *D *= 1835 accounting for 36.7% of *N *= 5000. The point estimate of the total number of expressed genes was N^
 MathType@MTEF@5@5@+=feaafiart1ev1aaatCvAUfKttLearuWrP9MDH5MBPbIqV92AaeXatLxBI9gBaebbnrfifHhDYfgasaacH8akY=wiFfYdH8Gipec8Eeeu0xXdbba9frFj0=OqFfea0dXdd9vqai=hGuQ8kuc9pgc9s8qqaq=dirpe0xb9q8qiLsFr0=vr0=vr0dc8meaabaqaciGacaGaaeqabaqabeGadaaakeaacuWGobGtgaqcaaaa@2DE3@ = 5023 with 95% bootstrap confidence interval (3617, 5492). With the initial sample fixed, we had resumed sampling of additional 1500, 3000, 4500 and 6000 ESTs (corresponding to time *t *= 0.5, 1, 1.5, 2), 200 times for each. The actual capture of additional new genes was recorded for each sample at each *t*. The sample mean of the 200 Monte Carlo estimates was used to approximate the true conditional mean *E*(*D*_*t*_|*D*) below (Note: the Monte Carlo mean for *D*_*t*_|*D *based on 200 samples is an accurate estimate for *E*(*D*_*t*_|*D*) since *D*_*t*_|*D *follows a Binomial distribution (Equation (1)).

Our method predicted that about 495, 870, 1171 and 1421 additional distinct genes would be expected to capture in these additional samples with 95% confidence intervals for *E*(*D*_*t*_|*D*) as (470, 514), (801, 908), (1043, 1227) and (1223, 1501) respectively, which well covered the corresponding expected conditional mean 502, 876, 1168 and 1403.

Though the SR method in *egene *was defined for *E*(*D*_*t*_), in EST sequencing one intends to use it to produce approximate estimates of the conditional capture *E*(*D*_*t*_|*D*), which is of direct interest given the current EST sample. The point estimates and corresponding standard errors (in parentheses below) of *E*(*D*_*t*_) from *egene *were 501 (17.63), 889 (42.67), 1128 (144.96), 244 (1333.8) at *t *= 0.5,1,1.5,2 with 95% confidence intervals (calculated based on E(Dt)_
 MathType@MTEF@5@5@+=feaafiart1ev1aaatCvAUfKttLearuWrP9MDH5MBPbIqV92AaeXatLxBI9gBaebbnrfifHhDYfgasaacH8akY=wiFfYdH8Gipec8Eeeu0xXdbba9frFj0=OqFfea0dXdd9vqai=hGuQ8kuc9pgc9s8qqaq=dirpe0xb9q8qiLsFr0=vr0=vr0dc8meaabaqaciGacaGaaeqabaqabeGadaaakeaadaqiaaqaaiabdweafnaabmaabaGaemiraq0aaSbaaSqaaiabdsha0bqabaaakiaawIcacaGLPaaaaiaawkWaaaaa@32C4@ ±1.96* *standard error*) are (466,536), (805,973), (844,1412) and (0,2857) respectively. We set the lower limit of the last confidence interval as zero because *E*(*D*_*t*_) must be greater than zero. The point estimate at *t *= 2 from the SR method was 244; this was unreasonable because it predicted fewer genes at t = 2 than at t = 0.5.

The second example was generated from setting (II) with S = 6000 and gene cluster profile data **n **= (n_1_...*n*_10_) = (2349, 888, 321,133,50,11,5,1,1,1). The total of sampled genes was *D *= 3760, accounting for 37.6% of *N*. The estimated total number of expressed genes was 8185 with 95% bootstrap confidence interval (7455,10441).

Our model predicted that with additional samples of size 3000, 6000, 9000 and 12000, we would expect to capture 991, 1715, 2266 and 2697 distinct genes with 95% confidence intervals (954,1005), (1626,1761), (2118, 2375) and (2479, 2884) respectively, again well covering the expected conditional capture 988, 1699, 2238 and 2660.

The *egene *program gave the point estimates of *E*(*D*_*t*_) and standard errors (in the parentheses) as 986 (25.4), 1692 (61.3), 2158 (202.8) and -718 (4082), corresponding to 95% confidence intervals (936,1036), (1572,1812) (1761, 2555) and (0, 7446) (for the same reason as in the first example, the lower limit of the last interval was set as 0).

The two case studies are typical among many simulations we have conducted, where the abundance distribution was highly rightward skewed and only a small fraction of the genes were captured in the initial EST sample. Based on our experience, we found that the bootstrap confidence interval for *E*(*D*_*t*_|*D*) always well covered the true mean *E*(*D*_*t*_|*D*) (approximated by the mean of Monte Carlo samples in our simulations) for *t *≤ 2. Although the SR method was defined for *E*(*D*_*t*_), it can be used to provide approximate estimates for the conditional capture *E*(*D*_*t*_|*D*) for *t *≤ 1, but in general it cannot be recommended for *t *≥ 1.

### Real data

We now apply the proposed methods to four cDNA libraries of *Arabidopsis thaliana *including green silique (3' EST), 2–6 weeks above-ground organs (5', to be called ABGR), root (5') and flower bud (3') obtained from NCBI dbEST (available at Supplementary Material). All the four cDNA libraries were normalized and size-selected [36]. ESTs were clustered using CAP3 with an overlap rule *O *= 40 bp, identity rule *P *= 90% and other parameters left at default. For the ABGR and root data (5' ESTs), the observed cluster counts were ISO error corrected using the correction matrix *P*_10 _simulated from *Arabidopsis thaliana *EST data by [27] (see Supplementary materials). For the silique and flower bud sets (3'), the gene cluster profile **n **was directly summarized from the CAP3 clustering results. The **n **data and the estimated number of expressed genes for these four sets are presented in Table 2 (complete list of the gene cluster profile data **n **can be found in the Supplementary Materials).

**Table 2:** Number of expressed genes in four cDNA libraries of *Arabidopsis thaliana*. This table lists the gene cluster profile data (n_j_), EST sample size(*EST.total*), observed gene number (*Gene.obsvd*), estimated total number of expressed genes (*Gene.estd*) and 95% confidence interval (95% *C.I*.) for 4 EST sets including Silique, ABGR, Root, Flower bud; and 2 pooled sets including ABGR + Root (A+R), Silique + Flower bud (S+F).

*n*_*j*_	Silique	ABGR	Root	Flower bud	A+R	S+F
*n*_1_	2963	1969	2187	1801	3333	3749
*n*_2_	994	459	490	367	951	1270
*n*_3_	440	182	133	140	312	566
*n*_4_	222	69	121	69	211	295
*n*_5_	124	58	37	40	122	182
*n*_6_	73	28	51	25	66	109
*n*_7_	59	17	22	22	40	80
*n*_8_	42	20	19	10	35	49
*n*_9_	27	7	7	15	29	48
*n*_10_	19	19	8	12	25	33
n11+ MathType@MTEF@5@5@+=feaafiart1ev1aaatCvAUfKttLearuWrP9MDH5MBPbIqV92AaeXatLxBI9gBaebbnrfifHhDYfgasaacH8akY=wiFfYdH8Gipec8Eeeu0xXdbba9frFj0=OqFfea0dXdd9vqai=hGuQ8kuc9pgc9s8qqaq=dirpe0xb9q8qiLsFr0=vr0=vr0dc8meaabaqaciGacaGaaeqabaqabeGadaaakeaacqWGUbGBdaqhaaWcbaGaeGymaeJaeGymaedabaGaey4kaScaaaaa@3102@	130	55	51	63	119	214

*EST.total*	12330	5812	5891	5503	11529	17784
*Gene.obsvd*	5093	2883	3126	2564	5243	6595
*Gene.estd*	12005	9492	9155	9232	12720	15333
95% C.I.	(11137,15300)	(7823,11585)	(8160,11444)	(7780,11381)	(11987,15579)	(13202,17400)

The results in Table 2 suggest that about 12005 genes were present in the green silique tissue library, in contrast to 9492, 9155 and 9232 in the ABGR, root and flower bud cDNA libraries respectively. It is possible that the green silique expressed more genes than the other three. However we lack confidence to conclude this because library screening (e.g., size selection) may cause such difference; in addition, under-estimation is likely in the latter three sets because of relatively small sample size. The 95% bootstrap confidence intervals for the four data sets were (11137,15300), (7823,11585), (8160,11444) and (7780,11381) respectively, which also failed to support the significance of the difference.

**Figure 2:**
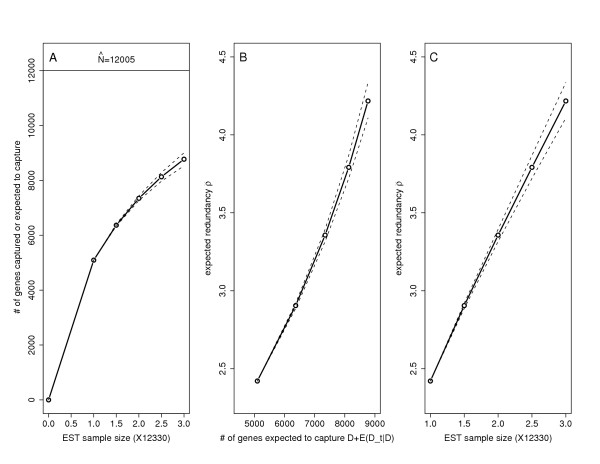
**Gene capture and redundancy prediction for green silique data. **The estimate of the total number of expressed genes is N^
 MathType@MTEF@5@5@+=feaafiart1ev1aaatCvAUfKttLearuWrP9MDH5MBPbIqV92AaeXatLxBI9gBaebbnrfifHhDYfgasaacH8akY=wiFfYdH8Gipec8Eeeu0xXdbba9frFj0=OqFfea0dXdd9vqai=hGuQ8kuc9pgc9s8qqaq=dirpe0xb9q8qiLsFr0=vr0=vr0dc8meaabaqaciGacaGaaeqabaqabeGadaaakeaacuWGobGtgaqcaaaa@2DE3@ = 12005. Plot (A) shows how the expected gene capture *E*(*D*_*t*_|*D*) with 95% confidence limits would increase with EST sample size; plots (B) and (C) show how the expected EST redundancy ρ_1+*t *_would increase with the expected gene capture (= *D *+ *E*(*D*_*t*_|*D*)) and EST sample size (= (1 + *t*)*S*)

In practice, the prediction is often made for sequencing in the near future, for example, for *t *≤ 2 (sequencing an additional ≤ 2*S *ESTs where *S *is the original sample size). In this situation the prediction can be adequately accurate even if bias exists for N^
 MathType@MTEF@5@5@+=feaafiart1ev1aaatCvAUfKttLearuWrP9MDH5MBPbIqV92AaeXatLxBI9gBaebbnrfifHhDYfgasaacH8akY=wiFfYdH8Gipec8Eeeu0xXdbba9frFj0=OqFfea0dXdd9vqai=hGuQ8kuc9pgc9s8qqaq=dirpe0xb9q8qiLsFr0=vr0=vr0dc8meaabaqaciGacaGaaeqabaqabeGadaaakeaacuWGobGtgaqcaaaa@2DE3@ based on our experience (see more in Discussion). We now use the green silique, ABGR, root and flower bud data to predict gene capture in the additional samples of size 0.5*S*, 1*S*, 1.5*S *and 2*S *(or *t *= 0.5,1,1.5,2, note: *S *is different for different EST sets). The results are presented in Table 3. In Figure 2, we plot gene capture (*D *+ E(Dt|D)_
 MathType@MTEF@5@5@+=feaafiart1ev1aaatCvAUfKttLearuWrP9MDH5MBPbIqV92AaeXatLxBI9gBaebbnrfifHhDYfgasaacH8akY=wiFfYdH8Gipec8Eeeu0xXdbba9frFj0=OqFfea0dXdd9vqai=hGuQ8kuc9pgc9s8qqaq=dirpe0xb9q8qiLsFr0=vr0=vr0dc8meaabaqaciGacaGaaeqabaqabeGadaaakeaadaqiaaqaaiabdweafnaabmaabaGaemiraq0aaSbaaSqaaiabdsha0bqabaGccqGG8baFcqWGebaraiaawIcacaGLPaaaaiaawkWaaaaa@3555@) versus EST sample size ((1 +*t*) *S), expected redundancy (ρ^1+t
 MathType@MTEF@5@5@+=feaafiart1ev1aaatCvAUfKttLearuWrP9MDH5MBPbIqV92AaeXatLxBI9gBaebbnrfifHhDYfgasaacH8akY=wiFfYdH8Gipec8Eeeu0xXdbba9frFj0=OqFfea0dXdd9vqai=hGuQ8kuc9pgc9s8qqaq=dirpe0xb9q8qiLsFr0=vr0=vr0dc8meaabaqaciaacaGaaeqabaqabeGadaaakeaacuaHbpGCgaqcamaaBaaaleaacqaIXaqmcqGHRaWkcqWG0baDaeqaaaaa@31EB@) versus expected gene capture (*D *+ E(Dt|D)_
 MathType@MTEF@5@5@+=feaafiart1ev1aaatCvAUfKttLearuWrP9MDH5MBPbIqV92AaeXatLxBI9gBaebbnrfifHhDYfgasaacH8akY=wiFfYdH8Gipec8Eeeu0xXdbba9frFj0=OqFfea0dXdd9vqai=hGuQ8kuc9pgc9s8qqaq=dirpe0xb9q8qiLsFr0=vr0=vr0dc8meaabaqaciGacaGaaeqabaqabeGadaaakeaadaqiaaqaaiabdweafnaabmaabaGaemiraq0aaSbaaSqaaiabdsha0bqabaGccqGG8baFcqWGebaraiaawIcacaGLPaaaaiaawkWaaaaa@3555@) and expected redundancy versus EST sample size ((1+t)*S) for the green silique (results are similar for the other three sets).

**Table 3:** Prediction of gene capture in an additional sample of size 0.5S, 1S, 1.5S and 2S. This table presents the estimates of *E*(*D*_*t*_|*D*) in additional samples of size 0.5S, 1S, 1.5S and 2S (or *t *= 0.5,1,1.5,2) with 95% bootstrap confidence interval(in the parentheses), where *S *is the sample size of original EST samples.

	0.5S	1S	1.5S	2S
Silique	1274 (1235,1302)	2253 (2159,2328)	3037 (2878,3172)	3678 (3450,3873)
ABGR	883 (854,906)	1616 (1540,1674)	2238 (2106,2345)	2776 (2577,2941)
Root	989(964,1011)	1806 (1737,1863)	2488(2363,2611)	3060(2871,3256)
Flower	820 (795,837)	1518(1453,1557)	2126 (2009,2198)	2659 (2480,2781)

For the silique data, if an additional sample of 12330 ESTs (*t *= 1) was sequenced, we would expect to capture an extra of 2253 distinct genes. The average gene capture per EST in the second sample is 0.18(= 2253/12330). For the ABGR, root and flower bud sets, this quantity (at *t *= 1) is 0.28, 0.31 and 0.28 respectively. The gene capture plot for the silique in Figure 2A shows a concave pattern in EST sample size, indicating an expected declining trend of efficiency with additional sequencing. The sequencing redundancy, defined as the average EST count per gene shows a slightly convex relationship in gene capture (Figure 2B) and a roughly linear one in EST sample size (Figure 2C). Note that these four cDNA libraries were generated under the same normalization protocol [36]; for non-normalized libraries, the redundancy would likely have increased at a greater rate as sequencing proceeded.

Now we turn to estimation of the number of genes jointly expressed or co-expressed in two pairs of tissues: silique + flower (3') and ABGR + root (5'). If we let *D*_1_, *D*_2 _and *D*_1∪2 _be the observed total number of genes in library 1, 2 and the pooled set, then the number of observed co-expressed genes is *D*_1∩2 _= *D*_1_+*D*_2_-*D*_1∪2_, in analogy with the estimated overlap N^1∩2=N^1+N^2−N^1∪2
 MathType@MTEF@5@5@+=feaafiart1ev1aaatCvAUfKttLearuWrP9MDH5MBPbIqV92AaeXatLxBI9gBaebbnrfifHhDYfgasaacH8akY=wiFfYdH8Gipec8Eeeu0xXdbba9frFj0=OqFfea0dXdd9vqai=hGuQ8kuc9pgc9s8qqaq=dirpe0xb9q8qiLsFr0=vr0=vr0dc8meaabaqaciaacaGaaeqabaqabeGadaaakeaacuWGobGtgaqcamaaBaaaleaacqaIXaqmcqGHPiYXcqaIYaGmaeqaaOGaeyypa0JafmOta4KbaKaadaWgaaWcbaGaeGymaedabeaakiabgUcaRiqbd6eaozaajaWaaSbaaSqaaiabikdaYaqabaGccqGHsislcuWGobGtgaqcamaaBaaaleaacqaIXaqmcqGHQicYcqaIYaGmaeqaaaaa@3E07@. The estimate of *N *in the silique and flower bud pair is 15333, suggesting an estimate of 5904 (= 9232+12005-15333) genes that are co-expressed in contrast to 1062 (= 5093+2564-6595) as observed. That is, about 64% (5904/9232) of the genes in flower bud tissue are actually co-expressed in the green silique tissue, much higher than 41% (1062/2564) as observed. For the second pair, the estimated total for the pooled set is 12720, suggesting an overlap of 5927 (= 9492+9155-12720) genes accounting for 65% of the total in the root tissue in contrast to 766 (= 2883+3126-5243) as observed for a fraction of 25%. Clearly the true between-library similarity in terms of the percentage of co-expressed genes is much higher than what is directly observed.

## Discussion

Several important factors could affect the accuracy and precision of gene capture prediction and gene number estimation. For applications of interest here, special care must first be taken to minimize the impact of errors from different sources. A good gene cluster profile data **n **should reflect the true sampling distribution of the transcripts in the cDNA library. We have suggested that investigators cluster 5' and 3' ESTs separately and then correct for errors attributable to insufficient overlap (ISO errors) of sibling 5' ESTs [27]. For the two 5' EST sets, root and ABGR, the estimates of *N *before and after ISO error correction were 12030 vs 9155 and 12085 vs 9492 respectively (see data before ISO error correction in the Supplementary Materials). The substantial difference in N^
 MathType@MTEF@5@5@+=feaafiart1ev1aaatCvAUfKttLearuWrP9MDH5MBPbIqV92AaeXatLxBI9gBaebbnrfifHhDYfgasaacH8akY=wiFfYdH8Gipec8Eeeu0xXdbba9frFj0=OqFfea0dXdd9vqai=hGuQ8kuc9pgc9s8qqaq=dirpe0xb9q8qiLsFr0=vr0=vr0dc8meaabaqaciGacaGaaeqabaqabeGadaaakeaacuWGobGtgaqcaaaa@2DE3@ is mainly due to the reduced singleton estimate (n^1
 MathType@MTEF@5@5@+=feaafiart1ev1aaatCvAUfKttLearuWrP9MDH5MBPbIqV92AaeXatLxBI9gBaebbnrfifHhDYfgasaacH8akY=wiFfYdH8Gipec8Eeeu0xXdbba9frFj0=OqFfea0dXdd9vqai=hGuQ8kuc9pgc9s8qqaq=dirpe0xb9q8qiLsFr0=vr0=vr0dc8meaabaqaciGacaGaaeqabaqabeGadaaakeaacuWGUbGBgaqcamaaBaaaleaacqaIXaqmaeqaaaaa@2F3F@) in the corrected version of gene cluster profile data n^
 MathType@MTEF@5@5@+=feaafiart1ev1aaatCvAUfKttLearuWrP9MDH5MBPbIqV92AaeXatLxBI9gBaebbnrfifHhDYfgasaacH8akY=wiFfYdH8Gipec8Eeeu0xXdbba9frFj0=OqFfea0dXdd9vqai=hGuQ8kuc9pgc9s8qqaq=dirpe0xb9q8qiLsFr0=vr0=vr0dc8meaabaqaciGacaGaaeqabaqabeGadaaakeaatCvAUfeBSjuyZL2yd9gzLbvyNv2CaeHbwvMCKfMBHbaceeGab8NBayaajaaaaa@3839@. In the gene capture prediction, we have treated n^
 MathType@MTEF@5@5@+=feaafiart1ev1aaatCvAUfKttLearuWrP9MDH5MBPbIqV92AaeXatLxBI9gBaebbnrfifHhDYfgasaacH8akY=wiFfYdH8Gipec8Eeeu0xXdbba9frFj0=OqFfea0dXdd9vqai=hGuQ8kuc9pgc9s8qqaq=dirpe0xb9q8qiLsFr0=vr0=vr0dc8meaabaqaciGacaGaaeqabaqabeGadaaakeaatCvAUfeBSjuyZL2yd9gzLbvyNv2CaeHbwvMCKfMBHbaceeGab8NBayaajaaaaa@3839@ as the true data for confidence inference. However estimating **n **itself by the ISO correction method could result in extra variability of predicted gene capture. This component of variability has not been taken into account in the bootstrap procedure.

Gene number estimation and gene capture prediction are sensitive to parametric assumptions of the transcript abundance distribution *Q*. A bad parametric assumption could yield a wildly biased estimate. For example, the Poisson-Gamma model due to Fisher [28] has been a popular choice in species number estimation problem, under which an analytical confidence interval can be obtained. However we found this assumption can yield extremely wild bias when the true *Q *deviates from Gamma [30]. The *egene *program by SR which implements the nonparametric empirical Bayes method by [34] and [29] has been shown unsatisfactory for prediction of additional gene capture *E*(*D*_*t*_) for *t *> 1 due to extreme variability. The Negative Binomial model discussed in [29] and [21] could potentially overcome the variability issue, however its performance has not been established in literature. We are unable to compare it with the CPP method since it is not integrated into *egene*.

The nonparametric maximum likelihood approach is typically robust to the form of transcript abundance distribution *Q*. For example, the gene capture prediction method worked remarkably well when *Q *was a log normal, exponential or gamma distribution. The nonparametric maximum likelihood estimator (NPMLE) of *Q*, i.e., Q^
 MathType@MTEF@5@5@+=feaafiart1ev1aaatCvAUfKttLearuWrP9MDH5MBPbIqV92AaeXatLxBI9gBaebbnrfifHhDYfgasaacH8akY=wiFfYdH8Gipec8Eeeu0xXdbba9frFj0=OqFfea0dXdd9vqai=hGuQ8kuc9pgc9s8qqaq=dirpe0xb9q8qiLsFr0=vr0=vr0dc8meaabaqaciGacaGaaeqabaqabeGadaaakeaacuWGrbqugaqcaaaa@2DE9@, provides a concise characterization of the transcript abundance distribution in the underlying cDNA library. In Theory the NPMLE Q^
 MathType@MTEF@5@5@+=feaafiart1ev1aaatCvAUfKttLearuWrP9MDH5MBPbIqV92AaeXatLxBI9gBaebbnrfifHhDYfgasaacH8akY=wiFfYdH8Gipec8Eeeu0xXdbba9frFj0=OqFfea0dXdd9vqai=hGuQ8kuc9pgc9s8qqaq=dirpe0xb9q8qiLsFr0=vr0=vr0dc8meaabaqaciGacaGaaeqabaqabeGadaaakeaacuWGrbqugaqcaaaa@2DE9@ is consistent for *Q *([37]), implying that Q^
 MathType@MTEF@5@5@+=feaafiart1ev1aaatCvAUfKttLearuWrP9MDH5MBPbIqV92AaeXatLxBI9gBaebbnrfifHhDYfgasaacH8akY=wiFfYdH8Gipec8Eeeu0xXdbba9frFj0=OqFfea0dXdd9vqai=hGuQ8kuc9pgc9s8qqaq=dirpe0xb9q8qiLsFr0=vr0=vr0dc8meaabaqaciGacaGaaeqabaqabeGadaaakeaacuWGrbqugaqcaaaa@2DE9@ will become adequately accurate in approximating *Q *as the sample size *S *is sufficiently large. For many EST libraries however, shallow sequencing provides little information of the rare genes. Consequently the NPMLE Q^
 MathType@MTEF@5@5@+=feaafiart1ev1aaatCvAUfKttLearuWrP9MDH5MBPbIqV92AaeXatLxBI9gBaebbnrfifHhDYfgasaacH8akY=wiFfYdH8Gipec8Eeeu0xXdbba9frFj0=OqFfea0dXdd9vqai=hGuQ8kuc9pgc9s8qqaq=dirpe0xb9q8qiLsFr0=vr0=vr0dc8meaabaqaciGacaGaaeqabaqabeGadaaakeaacuWGrbqugaqcaaaa@2DE9@ is often not accurate enough in characterizing the transcript abundance distribution at low levels. Thereby the number of rare genes was often under-estimated. The point estimate in the second simulated EST data set was N^
 MathType@MTEF@5@5@+=feaafiart1ev1aaatCvAUfKttLearuWrP9MDH5MBPbIqV92AaeXatLxBI9gBaebbnrfifHhDYfgasaacH8akY=wiFfYdH8Gipec8Eeeu0xXdbba9frFj0=OqFfea0dXdd9vqai=hGuQ8kuc9pgc9s8qqaq=dirpe0xb9q8qiLsFr0=vr0=vr0dc8meaabaqaciGacaGaaeqabaqabeGadaaakeaacuWGobGtgaqcaaaa@2DE3@ = 8185, appearing to be biased downward, though the bootstrap confidence interval covered the true *N*. For the ABGR, root and flower bud EST sets, we suspect that under-estimation exists owing to the relatively small sample size. Note in the CPP approach, N^
 MathType@MTEF@5@5@+=feaafiart1ev1aaatCvAUfKttLearuWrP9MDH5MBPbIqV92AaeXatLxBI9gBaebbnrfifHhDYfgasaacH8akY=wiFfYdH8Gipec8Eeeu0xXdbba9frFj0=OqFfea0dXdd9vqai=hGuQ8kuc9pgc9s8qqaq=dirpe0xb9q8qiLsFr0=vr0=vr0dc8meaabaqaciGacaGaaeqabaqabeGadaaakeaacuWGobGtgaqcaaaa@2DE3@ = *D *+ *lim*_*t*→∞_E(Dt|D)_
 MathType@MTEF@5@5@+=feaafiart1ev1aaatCvAUfKttLearuWrP9MDH5MBPbIqV92AaeXatLxBI9gBaebbnrfifHhDYfgasaacH8akY=wiFfYdH8Gipec8Eeeu0xXdbba9frFj0=OqFfea0dXdd9vqai=hGuQ8kuc9pgc9s8qqaq=dirpe0xb9q8qiLsFr0=vr0=vr0dc8meaabaqaciGacaGaaeqabaqabeGadaaakeaadaqiaaqaaiabdweafnaabmaabaGaemiraq0aaSbaaSqaaiabdsha0bqabaGccqGG8baFcqWGebaraiaawIcacaGLPaaaaiaawkWaaaaa@3555@. Even if N^
 MathType@MTEF@5@5@+=feaafiart1ev1aaatCvAUfKttLearuWrP9MDH5MBPbIqV92AaeXatLxBI9gBaebbnrfifHhDYfgasaacH8akY=wiFfYdH8Gipec8Eeeu0xXdbba9frFj0=OqFfea0dXdd9vqai=hGuQ8kuc9pgc9s8qqaq=dirpe0xb9q8qiLsFr0=vr0=vr0dc8meaabaqaciGacaGaaeqabaqabeGadaaakeaacuWGobGtgaqcaaaa@2DE3@ (at *t *→ ∞) were an under-estimate, the under-estimation effect would attenuate as *t *→ 0. Therefore for gene capture prediction in the near future (e.g. *t *≤ 2), the CPP method often works adequately well as shown in the second simulated EST set.

We have also demonstrated applications of the proposed method for estimating the number of expressed genes in one cDNA library or genes co-expressed in two libraries. The analysis of four EST data sets from normalized cDNA libraries of *Arabidopsis thaliana *disclosed a very similar concave pattern of gene capture together with a roughly linear increasing redundancy if sequencing had proceeded, both suggesting a rapid decay of sequencing efficiency. It seems to us that under-estimation is likely for *N *estimation if the EST sample size is relatively small. However the estimated gene expression overlap of two libraries still can be very informative for the true expression similarity provided the sample size is reasonably large.

The gene number estimation can be inflated if many genes have multiple splicing forms in the expression pool. ESTs from different splicing forms can fall into different contigs, causing an upwardly biased frequency of small clusters. In particular, the singleton count *n*_1 _will be inflated [27]. In general the singleton count is a sensitive indicator of the rare genes. Inflation of the singleton count *n*_1 _usually results in inflation of N^
 MathType@MTEF@5@5@+=feaafiart1ev1aaatCvAUfKttLearuWrP9MDH5MBPbIqV92AaeXatLxBI9gBaebbnrfifHhDYfgasaacH8akY=wiFfYdH8Gipec8Eeeu0xXdbba9frFj0=OqFfea0dXdd9vqai=hGuQ8kuc9pgc9s8qqaq=dirpe0xb9q8qiLsFr0=vr0=vr0dc8meaabaqaciGacaGaaeqabaqabeGadaaakeaacuWGobGtgaqcaaaa@2DE3@. If we had defined a "gene" as a distinct transcript, then this estimate will be biased downward because ESTs from different splicing forms of the same gene can fail to be distinguished in the clustering.

## Conclusion

We have proposed a compound Poisson process model for gene capture prediction and showed its superior performance over an existing approach in estimating the unconditional capture *E*(*D*_*t*_) by Monte Carlo simulations. We also showed its remarkable performance in predicting the future gene capture given the current EST sample. The analysis of four *Arabidopsis thaliana *EST sets showed that the number of expressed genes present in the parental cDNA libraries could vary from 7800 to 15000, while the fraction of co-expressed genes between two libraries can be much higher than the observed overlap. The approach can be used as a convenient, robust and reliable prediction tool in EST sequencing.

## Methods

### Estimating *Q*

To estimate *Q*, we adopt a penalized conditional nonparametric maximum likelihood (NPML) approach proposed in our previous work for species number estimation problem [30]. Note the likelihood in this problem can be written as

L(N,Q)=(Nn0,n1,...)∏j=0∞f(j;Q)nj∝(ND)f(0;Q)N−D[1−f(0;Q)]D×∏j>0∞[f(j;Q)1−f(0;Q)]nj≡Lm(N,Q)×Lc(Q),
 MathType@MTEF@5@5@+=feaafiart1ev1aaatCvAUfKttLearuWrP9MDH5MBPbIqV92AaeXatLxBI9gBaebbnrfifHhDYfgasaacH8akY=wiFfYdH8Gipec8Eeeu0xXdbba9frFj0=OqFfea0dXdd9vqai=hGuQ8kuc9pgc9s8qqaq=dirpe0xb9q8qiLsFr0=vr0=vr0dc8meaabaqaciaacaGaaeqabaqabeGadaaakeGabaafeuaabaqadiaaaeaacqWGmbatcqGGOaakcqWGobGtcqGGSaalcqWGrbqucqGGPaqkaeaacqGH9aqpdaqadaqaauaabeqaceaaaeaacqWGobGtaeaacqWGUbGBdaWgaaWcbaGaeGimaadabeaakiabcYcaSiabd6gaUnaaBaaaleaacqaIXaqmaeqaaOGaeiilaWIaeiOla4IaeiOla4IaeiOla4caaaGaayjkaiaawMcaamaarahabaGaemOzayMaeiikaGIaemOAaOMaei4oaSJaemyuaeLaeiykaKYaaWbaaSqabeaacqWGUbGBdaWgaaadbaGaemOAaOgabeaaaaaaleaacqWGQbGAcqGH9aqpcqaIWaamaeaacqGHEisPa0Gaey4dIunaaOqaaaqaaGGaaiab=1Hi1oaabmaabaqbaeqabiqaaaqaaiabd6eaobqaaiabdseaebaaaiaawIcacaGLPaaacqWGMbGzcqGGOaakcqaIWaamcqGG7aWocqWGrbqucqGGPaqkdaahaaWcbeqaaiabd6eaojabgkHiTiabdseaebaakiabcUfaBjabigdaXiabgkHiTiabdAgaMjabcIcaOiabicdaWiabcUda7iabdgfarjabcMcaPiabc2faDnaaCaaaleqabaGaemiraqeaaOGaey41aq7aaebCaeaadaWadaqaamaalaaabaGaemOzayMaeiikaGIaemOAaOMaei4oaSJaemyuaeLaeiykaKcabaGaeGymaeJaeyOeI0IaemOzayMaeiikaGIaeGimaaJaei4oaSJaemyuaeLaeiykaKcaaaGaay5waiaaw2faaaWcbaGaemOAaOMaeyOpa4JaeGimaadabaGaeyOhIukaniabg+GivdGcdaahaaWcbeqaaiabd6gaUnaaBaaameaacqWGQbGAaeqaaaaaaOqaaaqaaiabggMi6kabdYeamnaaBaaaleaacqWGTbqBaeqaaOGaeiikaGIaemOta4KaeiilaWIaemyuaeLaeiykaKIaey41aqRaemitaW0aaSbaaSqaaiabdogaJbqabaGccqGGOaakcqWGrbqucqGGPaqkcqGGSaalaaaaaa@99F2@

where *L*_*m*_(*N, Q*), is from the marginal distribution of *D*, depending on both *N *and *Q *and *L*_*c*_(*Q*) is from the conditional distribution of *X *given *D*, depending upon *Q *alone. Briefly the nonparametric MLE Q^
 MathType@MTEF@5@5@+=feaafiart1ev1aaatCvAUfKttLearuWrP9MDH5MBPbIqV92AaeXatLxBI9gBaebbnrfifHhDYfgasaacH8akY=wiFfYdH8Gipec8Eeeu0xXdbba9frFj0=OqFfea0dXdd9vqai=hGuQ8kuc9pgc9s8qqaq=dirpe0xb9q8qiLsFr0=vr0=vr0dc8meaabaqaciGacaGaaeqabaqabeGadaaakeaacuWGrbqugaqcaaaa@2DE9@ is first obtained based on the conditional likelihood *L*_*c*_(*Q*) modified by a penalty term which was designed to stabilize the estimation. A conditional MLE of *N *(N^WL
 MathType@MTEF@5@5@+=feaafiart1ev1aaatCvAUfKttLearuWrP9MDH5MBPbIqV92AaeXatLxBI9gBaebbnrfifHhDYfgasaacH8akY=wiFfYdH8Gipec8Eeeu0xXdbba9frFj0=OqFfea0dXdd9vqai=hGuQ8kuc9pgc9s8qqaq=dirpe0xb9q8qiLsFr0=vr0=vr0dc8meaabaqaciaacaGaaeqabaqabeGadaaakeaacuWGobGtgaqcamaaBaaaleaacqWGxbWvcqWGmbataeqaaaaa@3065@ in [30]) would be one that maximizes *L*_*m *_given Q^
 MathType@MTEF@5@5@+=feaafiart1ev1aaatCvAUfKttLearuWrP9MDH5MBPbIqV92AaeXatLxBI9gBaebbnrfifHhDYfgasaacH8akY=wiFfYdH8Gipec8Eeeu0xXdbba9frFj0=OqFfea0dXdd9vqai=hGuQ8kuc9pgc9s8qqaq=dirpe0xb9q8qiLsFr0=vr0=vr0dc8meaabaqaciGacaGaaeqabaqabeGadaaakeaacuWGrbqugaqcaaaa@2DE9@, which coincides with N^
 MathType@MTEF@5@5@+=feaafiart1ev1aaatCvAUfKttLearuWrP9MDH5MBPbIqV92AaeXatLxBI9gBaebbnrfifHhDYfgasaacH8akY=wiFfYdH8Gipec8Eeeu0xXdbba9frFj0=OqFfea0dXdd9vqai=hGuQ8kuc9pgc9s8qqaq=dirpe0xb9q8qiLsFr0=vr0=vr0dc8meaabaqaciGacaGaaeqabaqabeGadaaakeaacuWGobGtgaqcaaaa@2DE3@ from the Poisson process model proposed here, i.e. in the extrapolation form Dq_1
 MathType@MTEF@5@5@+=feaafiart1ev1aaatCvAUfKttLearuWrP9MDH5MBPbIqV92AaeXatLxBI9gBaebbnrfifHhDYfgasaacH8akY=wiFfYdH8Gipec8Eeeu0xXdbba9frFj0=OqFfea0dXdd9vqai=hGuQ8kuc9pgc9s8qqaq=dirpe0xb9q8qiLsFr0=vr0=vr0dc8meaabaqaciGacaGaaeqabaqabeGadaaakeaadaWcaaqaaiabdseaebqaamaaHaaabaGaemyCaehacaGLcmaadaWgaaWcbaGaeGymaedabeaaaaaaaa@3118@. From this perspective, the compound Poisson process model can be regarded as a generalization or extension of the mixture model in [30]. Details of Q^
 MathType@MTEF@5@5@+=feaafiart1ev1aaatCvAUfKttLearuWrP9MDH5MBPbIqV92AaeXatLxBI9gBaebbnrfifHhDYfgasaacH8akY=wiFfYdH8Gipec8Eeeu0xXdbba9frFj0=OqFfea0dXdd9vqai=hGuQ8kuc9pgc9s8qqaq=dirpe0xb9q8qiLsFr0=vr0=vr0dc8meaabaqaciGacaGaaeqabaqabeGadaaakeaacuWGrbqugaqcaaaa@2DE9@ estimation and remarkable performance of N^
 MathType@MTEF@5@5@+=feaafiart1ev1aaatCvAUfKttLearuWrP9MDH5MBPbIqV92AaeXatLxBI9gBaebbnrfifHhDYfgasaacH8akY=wiFfYdH8Gipec8Eeeu0xXdbba9frFj0=OqFfea0dXdd9vqai=hGuQ8kuc9pgc9s8qqaq=dirpe0xb9q8qiLsFr0=vr0=vr0dc8meaabaqaciGacaGaaeqabaqabeGadaaakeaacuWGobGtgaqcaaaa@2DE3@ are referred to [30].

### Confidence inference

Since in the NPML estimation, analytical confidence interval is not obtainable, we construct the confidence interval for *N, E*(*D*_*t*_|*D*) and ρ_1+*t *_by a bootstrap procedure. Since *D *is fixed in the conditional capture estimation, for each bootstrap sample, we would like to create *D *non-zero observations from the Poisson mixture distribution *f*(*x*;Q^
 MathType@MTEF@5@5@+=feaafiart1ev1aaatCvAUfKttLearuWrP9MDH5MBPbIqV92AaeXatLxBI9gBaebbnrfifHhDYfgasaacH8akY=wiFfYdH8Gipec8Eeeu0xXdbba9frFj0=OqFfea0dXdd9vqai=hGuQ8kuc9pgc9s8qqaq=dirpe0xb9q8qiLsFr0=vr0=vr0dc8meaabaqaciGacaGaaeqabaqabeGadaaakeaacuWGrbqugaqcaaaa@2DE9@) (discard zeroes from *f*(*0*;Q^
 MathType@MTEF@5@5@+=feaafiart1ev1aaatCvAUfKttLearuWrP9MDH5MBPbIqV92AaeXatLxBI9gBaebbnrfifHhDYfgasaacH8akY=wiFfYdH8Gipec8Eeeu0xXdbba9frFj0=OqFfea0dXdd9vqai=hGuQ8kuc9pgc9s8qqaq=dirpe0xb9q8qiLsFr0=vr0=vr0dc8meaabaqaciGacaGaaeqabaqabeGadaaakeaacuWGrbqugaqcaaaa@2DE9@) or directly simulate *D *observations from the zero-truncated Poisson mixture, i.e. f(x;Q^)1−f(0;Q^)
 MathType@MTEF@5@5@+=feaafiart1ev1aaatCvAUfKttLearuWrP9MDH5MBPbIqV92AaeXatLxBI9gBaebbnrfifHhDYfgasaacH8akY=wiFfYdH8Gipec8Eeeu0xXdbba9frFj0=OqFfea0dXdd9vqai=hGuQ8kuc9pgc9s8qqaq=dirpe0xb9q8qiLsFr0=vr0=vr0dc8meaabaqaciaacaGaaeqabaqabeGadaaakeaadaWcaaqaaiabdAgaMjabcIcaOiabdIha4jabcUda7iqbdgfarzaajaGaeiykaKcabaGaeGymaeJaeyOeI0IaemOzayMaeiikaGIaeGimaaJaei4oaSJafmyuaeLbaKaacqGGPaqkaaaaaa@3B80@ for *x *= 1,2...). Ideally one would also like to fix the bootstrap EST sample size (i.e. S(b)≡∑i=1DXi
 MathType@MTEF@5@5@+=feaafiart1ev1aaatCvAUfeBSjuyZL2yd9gzLbvyNv2CaerbwvMCKfMBHbqedmvETj2BSbqee0evGueE0jxyaibaieYdOi=BH8vipeYdI8qiW7rqqrFfpeea0xe9Lq=Jc9vqaqpepm0xbbG8FasPYRqj0=yi0lXdbba9pGe9qqFf0dXdHuk9fr=xfr=xfrpiWZqaaeaabiGaciaacaqabeaabeqacmaaaOqaaiaadofadaahaaWcbeqaaiaacIcacaWGIbGaaiykaaaakiabggMi6oaaqadabaGaamiwamaaBaaaleaacaWGPbaabeaaaeaacaWGPbGaeyypa0JaaGymaaqaaiaadseaa0GaeyyeIuoaaaa@3C42@) at *S *such that each sample strictly corresponds to a Poisson process at time interval [0, 1] as defined earlier. The bootstrap sample size *S*^(*b*) ^however, is a random variable and the sampling probability at *S*, i.e. *Prob*(*S*^(*b*) ^= *S*) is usually close to 0. We propose realizing this approximately by choosing bootstrap samples of size close to *S*, i.e. |*S*^(b) ^- *S*| ≤ *T *for some small integer *T*, e.g. T = 5 was used throughout this paper. Bootstrap samples were repeatedly generated until a total of 200 satisfying this constraint were obtained. For the *b*th sample, we obtain N^(b),E(Dt(b)|D_)
 MathType@MTEF@5@5@+=feaafiart1ev1aaatCvAUfKttLearuWrP9MDH5MBPbIqV92AaeXatLxBI9gBaebbnrfifHhDYfgasaacH8akY=wiFfYdH8Gipec8Eeeu0xXdbba9frFj0=OqFfea0dXdd9vqai=hGuQ8kuc9pgc9s8qqaq=dirpe0xb9q8qiLsFr0=vr0=vr0dc8meaabaqaciaacaGaaeqabaqabeGadaaakeaacuWGobGtgaqcamaaCaaaleqabaGaeiikaGIaemOyaiMaeiykaKcaaOGaeiilaWIaemyrauKaeiikaGYaaecaaeaacqWGebardaqhaaWcbaGaemiDaqhabaGaeiikaGIaemOyaiMaeiykaKcaaOWaaqqaaeaacqWGebaraiaawEa7aaGaayPadaGaeiykaKcaaa@3DDB@ and ρ^1+t(b)
 MathType@MTEF@5@5@+=feaafiart1ev1aaatCvAUfKttLearuWrP9MDH5MBPbIqV92AaeXatLxBI9gBaebbnrfifHhDYfgasaacH8akY=wiFfYdH8Gipec8Eeeu0xXdbba9frFj0=OqFfea0dXdd9vqai=hGuQ8kuc9pgc9s8qqaq=dirpe0xb9q8qiLsFr0=vr0=vr0dc8meaabaqaciaacaGaaeqabaqabeGadaaakeaaiiaacuWFbpGCgaqcamaaDaaaleaacqaIXaqmcqGHRaWkcqWG0baDaeaacqGGOaakcqWGIbGycqGGPaqkaaaaaa@34F0@ for *b *= 1, ... 200. The confidence interval for each quantity is constructed using Efron's percentile method [38].

### Joint expression estimation

In some situations, the number of genes jointly expressed in multiple tissues is also of interest. For example, one might want to know how many genes are expressed in an organ that has been sampled repeatedly, or at different developmental stages. Our method can be directly applied to estimate this quantity by pooling multiple EST sets. If the expression of gene *i *in the *j*th library, *X*_*ij *_follows a Poisson process with mean rate λ_*ij*_, then the total number of observed ESTs for this gene across *J *libraries, namely ∑j=1JXij
 MathType@MTEF@5@5@+=feaafiart1ev1aaatCvAUfKttLearuWrP9MDH5MBPbIqV92AaeXatLxBI9gBaebbnrfifHhDYfgasaacH8akY=wiFfYdH8Gipec8Eeeu0dXdbba9frFj0=OqFfea0dXdd9vqai=hGuQ8kuc9pgc9s8qqaq=dirpe0xb9q8qiLsFr0=vr0=vr0dc8meaabaqaciaacaGaaeqabaqabeGadaaakeaadaaeWaqaaiabdIfaynaaBaaaleaacqWGPbqAcqWGQbGAaeqaaaqcbawaaiabdQgaQjabg2da9iabigdaXaqaaiabdQeakbqdcqGHris5aaaa@3795@, will also follow a Poisson with pooled mean ∑j=1Jλij
 MathType@MTEF@5@5@+=feaafiart1ev1aaatCvAUfKttLearuWrP9MDH5MBPbIqV92AaeXatLxBI9gBaebbnrfifHhDYfgasaacH8akY=wiFfYdH8Gipec8Eeeu0dXdbba9frFj0=OqFfea0dXdd9vqai=hGuQ8kuc9pgc9s8qqaq=dirpe0xb9q8qiLsFr0=vr0=vr0dc8meaabaqaciaacaGaaeqabaqabeGadaaakeaadaaeWaqaaiabeU7aSnaaBaaaleaacqWGPbqAcqWGQbGAaeqaaaqcbawaaiabdQgaQjabg2da9iabigdaXaqaaiabdQeakbqdcqGHris5aaaa@3810@given that *X*_*ij *_are independent across *j*. Hence we can still model the gene cluster profile in the joint set with a Poisson mixture.

### Overlap expression estimation

We now consider to estimate the number of genes co-expressed in two libraries, say *L*_1 _and *L*_2_. Let *X*_*i *_= *X*_*i1*_+*X*_*i*2 _be the observed count of ESTs from the *i*th gene in the pooled set, and *X*_*ij *_be that from EST set *j*, for *j *= 1, 2. If the joint expression profile *X*_*ij *_can be accurately obtained (without clustering error), one could apply the method by [39] to estimate the number of co-expressed genes in two cDNA libraries. Unfortunately, because of clustering error, the observed *X*_*i*_, *X*_*ij *_can be inaccurate. For example, if we observe *X*_*i *_= *X*_*i*1_+*X*_*i*2 _= 3 + 4 = 7, then 7 can be separated from a larger cluster of size 8, 9, ..., due to insufficient overlap error in the 5' EST case [27]. Consequently, the observed *X*_*i*_, *X*_*ij *_all have measurement error, and must be corrected simultaneously. This could be quite complicated.

We here take an indirect way to tackle this problem. Suppose *N*_1 _and *N*_2 _are the numbers of genes present in cDNA library *L*_1 _and *L*_2 _respectively, and *N*_1∪2 _is the number of genes that are jointly expressed. Then the overlap of the two, denoted as *N*_1∩2_, can be expressed as:

*N*_1∩2 _= *N*_1_+ *N*_2 _- *N*_1∪2 _    (4)

For 5' ESTs, although the joint cluster profile *X*_*i *_= *X*_*i*1_+*X*_*i*2 _cannot be obtained accurately for all *i*, one can still obtain estimates of the marginal gene cluster profile for *L*_1_, *L*_2 _and *L*_1∪2 _separately in an unbiased fashion by the ISO correction method [27]. To do so, we first cluster ESTs within each library separately and then cluster the pooled set. One can obtain the ISO-error corrected gene cluster profiles n^1,n^2
 MathType@MTEF@5@5@+=feaafiart1ev1aaatCvAUfKttLearuWrP9MDH5MBPbIqV92AaeXatLxBI9gBaebbnrfifHhDYfgasaacH8akY=wiFfYdH8Gipec8Eeeu0xXdbba9frFj0=OqFfea0dXdd9vqai=hGuQ8kuc9pgc9s8qqaq=dirpe0xb9q8qiLsFr0=vr0=vr0dc8meaabaqaciaacaGaaeqabaqabeGadaaakeaaieqacuWFUbGBgaqcamaaBaaaleaacqaIXaqmaeqaaOGaeiilaWIaf8NBa4MbaKaadaWgaaWcbaGaeGOmaidabeaaaaa@32BC@ and n^1∪2
 MathType@MTEF@5@5@+=feaafiart1ev1aaatCvAUfKttLearuWrP9MDH5MBPbIqV92AaeXatLxBI9gBaebbnrfifHhDYfgasaacH8akY=wiFfYdH8Gipec8Eeeu0xXdbba9frFj0=OqFfea0dXdd9vqai=hGuQ8kuc9pgc9s8qqaq=dirpe0xb9q8qiLsFr0=vr0=vr0dc8meaabaqaciaacaGaaeqabaqabeGadaaakeaaieqacuWFUbGBgaqcamaaBaaaleaacqaIXaqmcqGHQicYcqaIYaGmaeqaaaaa@31D5@ and thereafter the estimates of gene number for these three sets, say N^1,N^2
 MathType@MTEF@5@5@+=feaafiart1ev1aaatCvAUfKttLearuWrP9MDH5MBPbIqV92AaeXatLxBI9gBaebbnrfifHhDYfgasaacH8akY=wiFfYdH8Gipec8Eeeu0xXdbba9frFj0=OqFfea0dXdd9vqai=hGuQ8kuc9pgc9s8qqaq=dirpe0xb9q8qiLsFr0=vr0=vr0dc8meaabaqaciaacaGaaeqabaqabeGadaaakeaacuWGobGtgaqcamaaBaaaleaacqaIXaqmaeqaaOGaeiilaWIafmOta4KbaKaadaWgaaWcbaGaeGOmaidabeaaaaa@323A@ and N^1∪2
 MathType@MTEF@5@5@+=feaafiart1ev1aaatCvAUfKttLearuWrP9MDH5MBPbIqV92AaeXatLxBI9gBaebbnrfifHhDYfgasaacH8akY=wiFfYdH8Gipec8Eeeu0xXdbba9frFj0=OqFfea0dXdd9vqai=hGuQ8kuc9pgc9s8qqaq=dirpe0xb9q8qiLsFr0=vr0=vr0dc8meaabaqaciGacaGaaeqabaqabeGadaaakeaacuWGobGtgaqcamaaBaaaleaacqaIXaqmcqGHQicYcqaIYaGmaeqaaaaa@3191@. A point estimate for *N*_1∩2 _would be

N^1∩2=N^1+N^2−N^1∪2.     (5)
 MathType@MTEF@5@5@+=feaafiart1ev1aaatCvAUfKttLearuWrP9MDH5MBPbIqV92AaeXatLxBI9gBaebbnrfifHhDYfgasaacH8akY=wiFfYdH8Gipec8Eeeu0xXdbba9frFj0=OqFfea0dXdd9vqai=hGuQ8kuc9pgc9s8qqaq=dirpe0xb9q8qiLsFr0=vr0=vr0dc8meaabaqaciaacaGaaeqabaqabeGadaaakeaacuWGobGtgaqcamaaBaaaleaacqaIXaqmcqGHPiYXcqaIYaGmaeqaaOGaeyypa0JafmOta4KbaKaadaWgaaWcbaGaeGymaedabeaakiabgUcaRiqbd6eaozaajaWaaSbaaSqaaiabikdaYaqabaGccqGHsislcuWGobGtgaqcamaaBaaaleaacqaIXaqmcqGHQicYcqaIYaGmaeqaaOGaeiOla4IaaCzcaiaaxMaacqGGOaakcqaI1aqncqGGPaqkaaa@42E3@

### Availability

The methods have been integrated into a web-based tool *EST stat*, which is available at [40]. The supplementary materials are also available at [41]. The current version of *EST stat *software provides two options for input file(s): (1) CAP3 clustering results including .*ace *and .*singlets *files; (2) the gene cluster profile data **n**. If the user chooses option (1), *ESTstat *will parse out the gene cluster profile data from CAP3 results; and for 5' ESTs, it will simulate ISO error and make ISO-error correction to generate n^
 MathType@MTEF@5@5@+=feaafiart1ev1aaatCvAUfKttLearuWrP9MDH5MBPbIqV92AaeXatLxBI9gBaebbnrfifHhDYfgasaacH8akY=wiFfYdH8Gipec8Eeeu0xXdbba9frFj0=OqFfea0dXdd9vqai=hGuQ8kuc9pgc9s8qqaq=dirpe0xb9q8qiLsFr0=vr0=vr0dc8meaabaqaciGacaGaaeqabaqabeGadaaakeaatCvAUfeBSjuyZL2yd9gzLbvyNv2CaeHbwvMCKfMBHbaceeGab8NBayaajaaaaa@3839@. If one has better gene cluster profile data **n**, he (she) can choose option (2) to obtain statistical analysis directly. Finding NPMLE is computationally intensive. The bootstrap function is currently not integrated into the web-based *EST stat *interface. A JAVA program is available at the Supplementary materials website allowing to obtain bootstrap confidence intervals for the total number of expressed genes, the additional capture and redundancy at the user-specified sample size.

## Authors' contributions

JW: development of methods and algorithms, data analysis, manuscript writing.

BL: development of statistical methods with JW, involved in manuscript writing.

LC: programming, web interface development, involved in manuscript writing.

PW: programming and *EST stat *maintenance.

JM: Perl script writing.

JZ: JAVA codes writing and simulation studies.

CD: project initialization, biological significance assessment, involved in manuscript writing.
